# 1,3-Diphenyl-8-trifluoro­methyl-1*H*-pyrazolo­[3,4-*b*]quinoline

**DOI:** 10.1107/S1600536812028206

**Published:** 2012-06-30

**Authors:** Paweł Szlachcic, Katarzyna Stadnicka

**Affiliations:** a Department of Chemistry and Physics, Agricultural University, 30-149 Kraków, Poland; b Faculty of Chemistry, Jagiellonian University, 30-060 Kraków, Poland

## Abstract

The 1*H*-pyrazolo­[3,4-*b*]quinoline (PQ) core of the title mol­ecule, C_23_H_14_F_3_N_3_, is aromatic and essentially planar (r.m.s. deviation = 0.015 Å) and the two phenyl substituents at positions 1 and 3 are twisted relative to this fragment by 29.74 (7) and 25.63 (7)°, respectively. In the crystal, mol­ecules are arranged along the *b* axis into stacks *via* π–π inter­actions, with an inter­planar distance of the PQ core of 3.489 (4) Å.

## Related literature

For selected photophysical properties of trifluoro­methyl derivatives of 1*H*-pyrazolo-[3,4-*b*]quinoline, see: Koścień, Gondek, Jarosz *et al.* (2009 ▶); Koścień, Gondek, Pokladko *et al.* (2009 ▶). For the use of trifluoro­methyl derivatives of 1*H*-pyrazolo-[3,4-*b*]quinoline in organic light-emitting diode (OLED) preparation, see: Tao *et al.* (2001 ▶). For the synthesis of 1*H*-pyrazolo-[3,4-*b*]quinoline derivatives, see: Brack (1965 ▶).
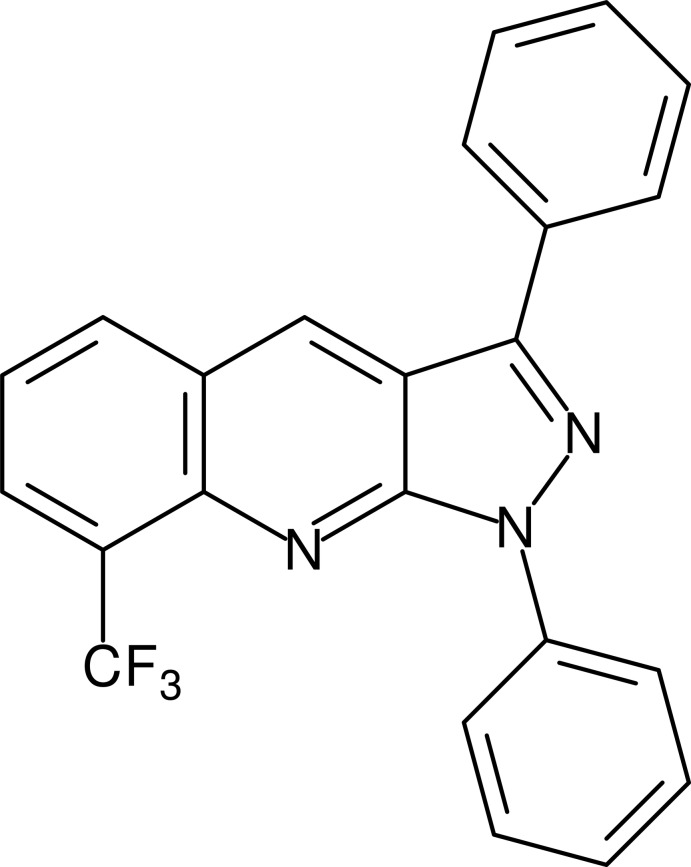


## Experimental

### 

#### Crystal data

C_23_H_14_F_3_N_3_
*M* *_r_* = 389.37
Monoclinic, 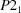

*a* = 11.8299 (5) Å
*b* = 6.9788 (3) Å
*c* = 12.1306 (4) Å
β = 112.765 (2)°
*V* = 923.47 (6) Å^3^
*Z* = 2
Mo *K*α radiation
μ = 0.11 mm^−1^
*T* = 293 K
0.27 × 0.25 × 0.20 mm

#### Data collection

Nonius KappaCCD diffractometer
Absorption correction: multi-scan (*DENZO* and *SCALEPACK*; Otwinowski & Minor, 1997 ▶) *T*_min_ = 0.972, *T*_max_ = 0.979
4497 measured reflections
4497 independent reflections
2183 reflections with *I* > 2σ(*I*)
*R*_int_ = 0.018

#### Refinement

*R*[*F* ^2^ > 2σ(*F* ^2^)] = 0.044
*wR*(*F* ^2^) = 0.104
*S* = 1.05
2876 reflections
262 parameters
1 restraint
H-atom parameters constrained
Δρ_max_ = 0.16 e Å^−3^
Δρ_min_ = −0.18 e Å^−3^

### 

Data collection: *COLLECT* (Nonius, 1998 ▶); cell refinement: *SCALEPACK* (Otwinowski & Minor, 1997 ▶); data reduction: *DENZO* (Otwinowski & Minor, 1997 ▶) and *SCALEPACK*; program(s) used to solve structure: *SIR92* (Altomare *et al.*, 1994 ▶); program(s) used to refine structure: *SHELXL97* (Sheldrick, 2008 ▶); molecular graphics: *ORTEP-3 for Windows* (Farrugia, 1997 ▶); software used to prepare material for publication: *SHELXL97*.

## Supplementary Material

Crystal structure: contains datablock(s) global, I. DOI: 10.1107/S1600536812028206/gk2493sup1.cif

Structure factors: contains datablock(s) I. DOI: 10.1107/S1600536812028206/gk2493Isup2.hkl

Supplementary material file. DOI: 10.1107/S1600536812028206/gk2493Isup3.cml

Additional supplementary materials:  crystallographic information; 3D view; checkCIF report

## Figures and Tables

**Fig. 1.**
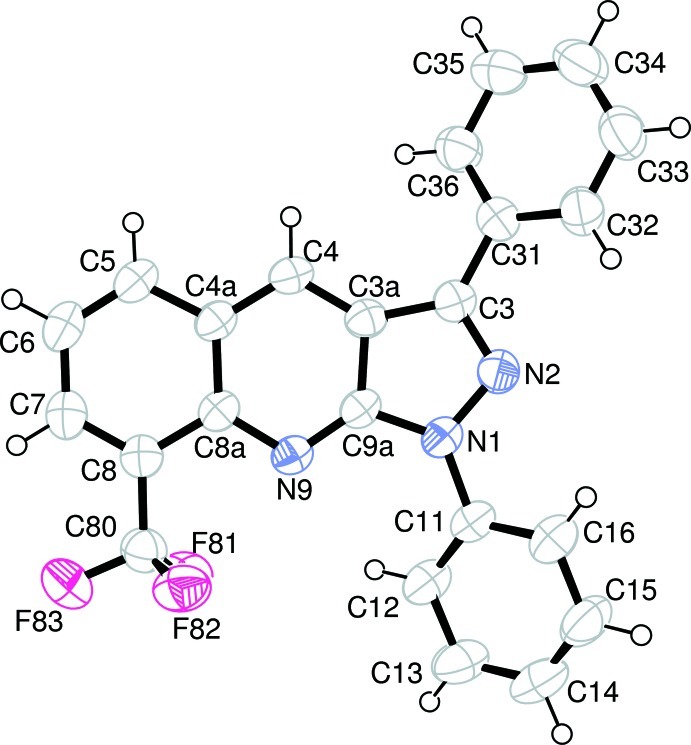
The best view of the molecule of title compound showing displacement ellipsoids at the 50% probability level.

**Fig. 2.**
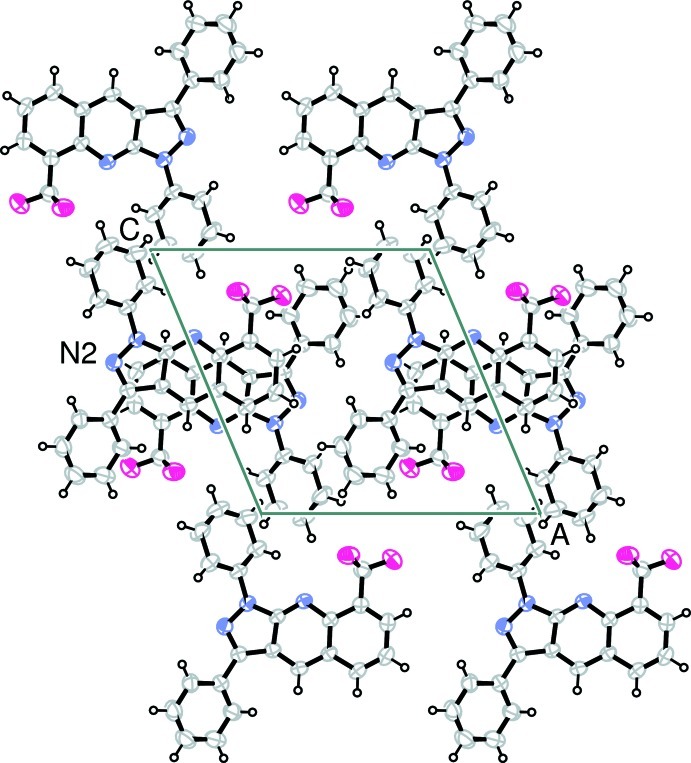
Projection of the unit-cell contents along [010]. The mutual arrangement of the molecules forming π–π interactions is illustrated. The unit cell origin is at the lower left-hand corner of the cell with b axis pointed down.

**Fig. 3.**
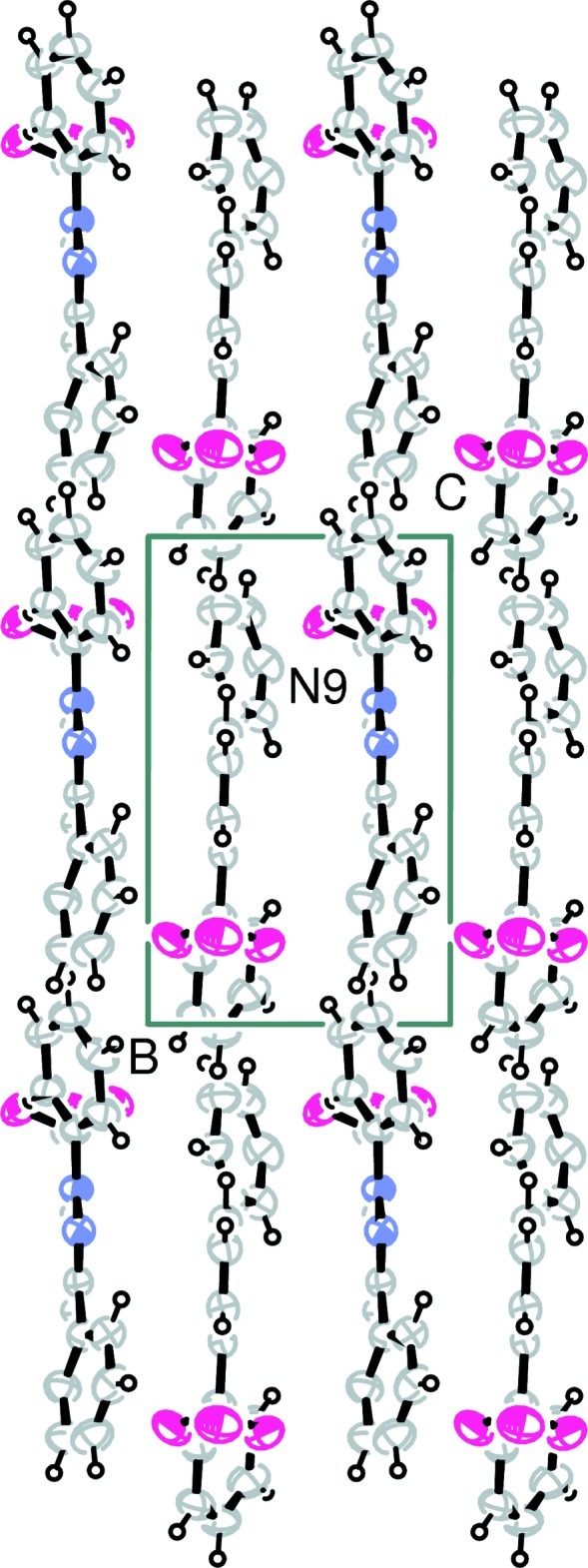
Projection of the unit-cell contents along [100] showing layered structure. The unit cell origin is at the lower right-hand corner of the cell with a axis pointed down.

## supplementary crystallographic information

### Comment

The derivatives of 1*H*-pyrazolo[3,4-*b*]quinoline (PQ) containing trifluoromethyl substituents at C7 were found to have interesting photophysical properties (Koścień, Gondek, Jarosz *et al.*, 2009; Koścień, Gondek, Pokladko *et al.*, 2009). A relatively high quantum efficiency allowed to propose the CF_3_ derivatives as blue-light luminophore and to use the derivatives as the chromophore for organic light-emitting diodes (OLED). To synthesize PQ derivatives with H atom in C-4 position, a known method of preparation was used (Brack, 1965). Previously it was found, that in the case of 7-trifluoromethyl-1-methyl-3-phenyl-1*H*-pyrazolo[3,4-*b*]quinoline, the incorporation of CF_3_ substituent into PQ molecule rises the values of HOMO/LUMO and ionization potential of the luminophore in comparison to PQ itself (Tao *et al.*, 2001), so 8-trifluoromethyl-1,3-diphenyl-1*H*-pyrazolo[3,4-*b*]quinoline was synthesized as the compound promising useful properties for the construction of OLED cells with Mg/Ag alloy kathode or even Al kathode. The results of using the trifluoromethyl derivatives of 1,3-diphenyl-1*H*-pyrazolo[3,4-*b*]quinoline for OLED preparation will be published elsewhere.

The shape of the title molecule is shown in Fig. 1. The core of the molecule, 1*H*-pyrazolo[3,4-*b*]quinoline, is planar and aromatic. Although the planes of both phenyl substituents should be coplanar with the core moiety (due to the conjugation between aromatic core and aromatic phenyl rings), they are slightly twisted with the torsion angles N2—N1—C11—C16 = 27.6 (4), N2—C3—C31—C32 = -23.9 (4)°. In the case of the phenyl substituent at C3 the effect is caused by the steric hindrance between the hydrogen atoms H4 and H36 (H4···H36 = 2.26 Å). The overall shape of the molecule is also influenced by weak intramolecular interaction C12—H12···N9 (Table 1). The trifluoromethyl group forms two hydrogen-bond-like intermolecular interactions of C—H···F type: intramolecular one C7—H7···F83 and intermolecular one C36—H36···F82 (-*x*, *y* + 1/2, -*z* + 1) with the geometrical parameters given in Table 1.

The packing of the molecules (Fig. 2 and Fig. 3) is determined mainly by intermolecular π–π stacking with the geometry given below (*Cg*···*Cg*···*Cg*/Å, <*CgCgCg* /°, respectively):

*Cg*3(C4a—C5—C6—C7—C8—C8a at -*x*, *y* - 1/2, -*z* + 1)···*Cg*1(N1—N2—C3—C3a—C9a)···*Cg*3(C4a—C5—C6—C7—C8—C8a at -*x*, *y* + 1/2, -*z* + 1): 3.751 (4), 3.906 (5), 131.4 (3);

*Cg*2(C3a—C4—C4a—C8a—N9—C9a at -*x*, *y* - 1/2, -*z* + 1)···*Cg*2(C3a—C4—C4a—C8a—N9—C9a)···*Cg*2(C3a—C4—C4a—C8a—N9—C9a at -*x*, *y* + 1/2, -*z* + 1): 3.799 (4), 3.799 (4), 133.5 (3);

*Cg*3(C4a—C5—C6—C7—C8—C8a at -*x*, *y* - 1/2, -*z* + 1)···*Cg*2(C3a—C4—C4a—C8a—N9—C9a)···*Cg*3(C4a—C5—C6—C7—C8—C8a at -*x*, *y* + 1/2, -*z* + 1): 3.732 (4), 3.787 (4), 136.3 (3).

The structure is additionaly stabilized by two C—H···π interactions: C13—H13···*Cg*4 (-x, y + 1/2, -z + 2) and C33—H33···*Cg*4 (-x - 1, y - 1/2, -z + 1/2) given in Table 1.

### Experimental

The title compound was synthesized using procedure already described in literature (Brack, 1965) from 2-(trifluoromethyl)aniline and 5-chloro-1,3-diphenyl-1*H*-pyrazol-4-carbaldehyde (5 mmol of each substrate, sulfolane as a solvent). The product was purified by column chromatography (SilicaGel 60, toluene/petroleum ether 1:1 as the eluent) followed by preparative TLC (SilicaGel 60, 2 mm, toluene/petroleum ether 1:1 as the eluent) to give 50 mg (2.6% yield - the low yield is caused by strong induction electron-withdrawing effect of the trifluoromethyl group in *ortho*-position to the amine group) of yellow crystalline solid, mp. 452–454 K. ^1^H NMR (CDCl_3_): δ 7.31 (tt, *J* = 7.4, 1.2 Hz, 1H), 7.48–7.63 (m, 6H), 8.13–8.19 (m, 4H), 8.74–8.77 (m, 2H), 8.95 (s, 1H); ^13^C NMR (CDCl_3_): δ 116.9, 119.9, 122.7, 124.8, 125.4, 127.5, 129.0, 129.1, 129.3, 129.6 (q, *J*_CF_ = 5.4 Hz), 131.3, 132.2, 133.7, 139.8, 144.0, 144.5, 150.1. Single crystals suitable for X-ray diffraction were grown by slow evaporation from toluene solution.

### Refinement

As the structure contains only C, H, N and F atoms Friedel pairs were merged and absolute structure was not determined. H atoms were included into refinement in geometrically calculated positions, with C—H = 0.93 Å, and *U*_iso_(H) = 1.2*U*_eq_( C) for the aromatic CH groups, and constrained as a part of a riding model.

### Crystal data

**Table:**

C_23_H_14_F_3_N_3_	*F*(000) = 400
*M**_r_* = 389.37	*D*_x_ = 1.400 Mg m^−^^3^
Monoclinic, *P*2_1_	Melting point = 452–454 K
Hall symbol: P 2yb	Mo *K*α radiation, λ = 0.71073 Å
*a* = 11.8299 (5) Å	Cell parameters from 2631 reflections
*b* = 6.9788 (3) Å	θ = 0.1–30.0°
*c* = 12.1306 (4) Å	µ = 0.11 mm^−^^1^
β = 112.765 (2)°	*T* = 293 K
*V* = 923.47 (6) Å^3^	Block, yellow
*Z* = 2	0.27 × 0.25 × 0.20 mm

### Data collection

**Table:**

Nonius KappaCCD diffractometer	4497 independent reflections
Radiation source: fine-focus sealed tube	2183 reflections with *I* > 2σ(*I*)
Horizontally mounted graphite crystal monochromator	*R*_int_ = 0.018
Detector resolution: 9 pixels mm^-1^	θ_max_ = 30.0°, θ_min_ = 3.1°
ω scans	*h* = −16→16
Absorption correction: multi-scan (*DENZO* and *SCALEPACK*; Otwinowski & Minor, 1997)	*k* = −8→9
*T*_min_ = 0.972, *T*_max_ = 0.979	*l* = −17→16
4497 measured reflections	

### Refinement

**Table:**

Refinement on *F*^2^	Primary atom site location: structure-invariant direct methods
Least-squares matrix: full	Secondary atom site location: difference Fourier map
*R*[*F*^2^ > 2σ(*F*^2^)] = 0.044	Hydrogen site location: inferred from neighbouring sites
*wR*(*F*^2^) = 0.104	H-atom parameters constrained
*S* = 1.05	*w* = 1/[σ^2^(*F*_o_^2^) + (0.0406*P*)^2^ + 0.1305*P*] where *P* = (*F*_o_^2^ + 2*F*_c_^2^)/3
2876 reflections	(Δ/σ)_max_ < 0.001
262 parameters	Δρ_max_ = 0.16 e Å^−^^3^
1 restraint	Δρ_min_ = −0.18 e Å^−^^3^
0 constraints	

### Special details

**Table:**

Geometry. All e.s.d.'s are estimated using the full covariance matrix. The cell e.s.d.'s are taken into account individually in the estimation of e.s.d.'s in distances, angles and torsion angles; correlations between e.s.d.'s in cell parameters are only used when they are defined by crystal symmetry.
Refinement. Refinement of *F*^2^ against all reflections. The weighted *R*-factor *wR* and goodness of fit *S* are based on *F*^2^, conventional *R*-factors *R* are based on *F*, with *F* set to zero for negative *F*^2^. The threshold expression of *F*^2^ > σ(*F*^2^) is used only for calculating *R*-factors(gt) *etc*. and is not relevant to the choice of reflections for refinement. *R*-factors based on *F*^2^ are statistically about twice as large as those based on *F*, and *R*- factors based on all data will be even larger.

### Fractional atomic coordinates and isotropic or equivalent isotropic displacement parameters (Å^2^)

**Table:**

	*x*	*y*	*z*	*U*_iso_*/*U*_eq_	
N1	−0.18386 (15)	0.2365 (4)	0.65903 (14)	0.0473 (4)	
N2	−0.30046 (15)	0.2283 (3)	0.57218 (15)	0.0491 (4)	
C3	−0.29086 (18)	0.2297 (4)	0.46736 (18)	0.0454 (5)	
C3A	−0.16388 (17)	0.2389 (4)	0.48271 (16)	0.0422 (4)	
C4	−0.09599 (18)	0.2371 (4)	0.41296 (17)	0.0453 (5)	
H4	−0.1342	0.2327	0.3301	0.054*	
C4A	0.03230 (18)	0.2421 (4)	0.46963 (16)	0.0432 (4)	
C5	0.1094 (2)	0.2426 (5)	0.40413 (18)	0.0518 (5)	
H5	0.0743	0.2407	0.3210	0.062*	
C6	0.2333 (2)	0.2456 (5)	0.4611 (2)	0.0572 (6)	
H6	0.2822	0.2441	0.4168	0.069*	
C7	0.28812 (19)	0.2510 (5)	0.5865 (2)	0.0535 (5)	
H7	0.3731	0.2536	0.6242	0.064*	
C8	0.21850 (18)	0.2525 (4)	0.65379 (17)	0.0455 (4)	
C8A	0.08787 (17)	0.2470 (4)	0.59753 (16)	0.0410 (4)	
N9	0.02219 (14)	0.2477 (3)	0.66801 (13)	0.0434 (4)	
C9A	−0.09735 (18)	0.2432 (4)	0.60926 (16)	0.0417 (4)	
C11	−0.16788 (19)	0.2393 (4)	0.78184 (17)	0.0477 (5)	
C12	−0.0697 (2)	0.3313 (4)	0.8651 (2)	0.0593 (7)	
H12	−0.0100	0.3868	0.8431	0.071*	
C13	−0.0599 (3)	0.3410 (5)	0.9832 (2)	0.0719 (8)	
H13	0.0068	0.4025	1.0405	0.086*	
C14	−0.1485 (3)	0.2596 (6)	1.0149 (2)	0.0756 (8)	
H14	−0.1428	0.2686	1.0934	0.091*	
C15	−0.2448 (3)	0.1658 (6)	0.9320 (3)	0.0766 (9)	
H15	−0.3035	0.1091	0.9549	0.092*	
C16	−0.2567 (3)	0.1534 (5)	0.8135 (2)	0.0639 (7)	
H16	−0.3226	0.0892	0.7570	0.077*	
C31	−0.40125 (19)	0.2166 (4)	0.3563 (2)	0.0486 (5)	
C32	−0.5081 (2)	0.1333 (5)	0.3575 (2)	0.0594 (6)	
H32	−0.5100	0.0878	0.4288	0.071*	
C33	−0.6107 (2)	0.1185 (5)	0.2531 (3)	0.0720 (8)	
H33	−0.6818	0.0641	0.2547	0.086*	
C34	−0.6094 (3)	0.1835 (5)	0.1461 (3)	0.0775 (10)	
H34	−0.6788	0.1721	0.0759	0.093*	
C35	−0.5043 (2)	0.2654 (7)	0.1446 (2)	0.0779 (9)	
H35	−0.5026	0.3098	0.0730	0.093*	
C36	−0.4008 (2)	0.2820 (5)	0.2494 (2)	0.0614 (7)	
H36	−0.3302	0.3380	0.2474	0.074*	
C80	0.2782 (2)	0.2633 (5)	0.7870 (2)	0.0567 (6)	
F81	0.24588 (17)	0.4210 (3)	0.83109 (15)	0.0736 (5)	
F82	0.25008 (17)	0.1139 (3)	0.84136 (15)	0.0723 (5)	
F83	0.40032 (12)	0.2662 (4)	0.82621 (13)	0.0813 (5)	

### Atomic displacement parameters (Å^2^)

**Table:**

	*U*^11^	*U*^22^	*U*^33^	*U*^12^	*U*^13^	*U*^23^
N1	0.0495 (9)	0.0539 (11)	0.0428 (8)	0.0010 (12)	0.0225 (7)	0.0000 (11)
N2	0.0477 (9)	0.0514 (11)	0.0502 (9)	0.0000 (11)	0.0211 (7)	0.0007 (11)
C3	0.0494 (10)	0.0415 (12)	0.0457 (10)	−0.0017 (12)	0.0190 (8)	0.0002 (11)
C3A	0.0477 (9)	0.0385 (10)	0.0405 (9)	−0.0031 (12)	0.0172 (8)	−0.0024 (11)
C4	0.0548 (11)	0.0457 (12)	0.0362 (8)	−0.0032 (13)	0.0183 (8)	−0.0025 (12)
C4A	0.0525 (10)	0.0392 (10)	0.0422 (9)	−0.0029 (12)	0.0231 (8)	−0.0025 (11)
C5	0.0632 (12)	0.0531 (13)	0.0481 (10)	−0.0026 (16)	0.0313 (10)	−0.0034 (15)
C6	0.0622 (13)	0.0584 (15)	0.0646 (13)	−0.0020 (17)	0.0395 (11)	−0.0050 (17)
C7	0.0488 (10)	0.0507 (13)	0.0651 (12)	−0.0026 (14)	0.0268 (10)	−0.0041 (15)
C8	0.0490 (10)	0.0386 (10)	0.0494 (10)	−0.0003 (12)	0.0195 (8)	−0.0034 (12)
C8A	0.0496 (10)	0.0335 (9)	0.0436 (9)	0.0001 (12)	0.0220 (8)	−0.0022 (11)
N9	0.0489 (8)	0.0445 (9)	0.0388 (7)	−0.0008 (11)	0.0191 (7)	−0.0026 (10)
C9A	0.0502 (10)	0.0388 (10)	0.0405 (9)	−0.0013 (12)	0.0223 (8)	−0.0018 (12)
C11	0.0608 (11)	0.0456 (11)	0.0433 (9)	0.0046 (14)	0.0274 (9)	0.0027 (13)
C12	0.0760 (16)	0.0589 (16)	0.0523 (13)	−0.0056 (14)	0.0351 (12)	−0.0023 (12)
C13	0.095 (2)	0.075 (2)	0.0485 (13)	−0.0102 (17)	0.0306 (14)	−0.0091 (14)
C14	0.106 (2)	0.084 (2)	0.0499 (12)	0.009 (2)	0.0448 (14)	0.0019 (17)
C15	0.0898 (19)	0.092 (2)	0.0646 (16)	−0.0012 (19)	0.0481 (16)	0.0157 (17)
C16	0.0735 (16)	0.0687 (18)	0.0587 (14)	−0.0023 (15)	0.0355 (13)	0.0074 (14)
C31	0.0464 (10)	0.0454 (14)	0.0520 (11)	0.0004 (11)	0.0169 (9)	−0.0019 (11)
C32	0.0515 (12)	0.0652 (16)	0.0629 (14)	−0.0039 (14)	0.0238 (11)	−0.0039 (14)
C33	0.0528 (14)	0.080 (2)	0.0799 (19)	−0.0115 (16)	0.0221 (13)	−0.0157 (18)
C34	0.0588 (15)	0.093 (3)	0.0647 (16)	−0.0047 (17)	0.0059 (12)	−0.0165 (17)
C35	0.0726 (16)	0.097 (3)	0.0521 (13)	−0.003 (2)	0.0114 (12)	0.0035 (19)
C36	0.0565 (12)	0.0682 (19)	0.0554 (13)	−0.0052 (14)	0.0170 (10)	0.0059 (13)
C80	0.0538 (12)	0.0606 (16)	0.0529 (11)	0.0031 (15)	0.0176 (10)	−0.0045 (14)
F81	0.0799 (12)	0.0745 (11)	0.0595 (10)	0.0008 (10)	0.0192 (9)	−0.0227 (9)
F82	0.0823 (12)	0.0769 (12)	0.0544 (9)	0.0061 (10)	0.0226 (9)	0.0131 (9)
F83	0.0526 (7)	0.1111 (15)	0.0676 (8)	−0.0001 (12)	0.0093 (6)	−0.0062 (12)

### Geometric parameters (Å, º)

**Table:**

N1—C9A	1.376 (2)	C12—C13	1.393 (3)
N1—N2	1.375 (2)	C12—H12	0.9300
N1—C11	1.427 (2)	C13—C14	1.372 (4)
N2—C3	1.320 (3)	C13—H13	0.9300
C3—C3A	1.442 (3)	C14—C15	1.361 (4)
C3—C31	1.472 (3)	C14—H14	0.9300
C3A—C4	1.374 (3)	C15—C16	1.392 (4)
C3A—C9A	1.429 (3)	C15—H15	0.9300
C4—C4A	1.403 (3)	C16—H16	0.9300
C4—H4	0.9300	C31—C36	1.378 (3)
C4A—C5	1.422 (3)	C31—C32	1.396 (3)
C4A—C8A	1.432 (3)	C32—C33	1.378 (4)
C5—C6	1.358 (3)	C32—H32	0.9300
C5—H5	0.9300	C33—C34	1.380 (5)
C6—C7	1.404 (3)	C33—H33	0.9300
C6—H6	0.9300	C34—C35	1.375 (4)
C7—C8	1.366 (3)	C34—H34	0.9300
C7—H7	0.9300	C35—C36	1.387 (3)
C8—C8A	1.428 (3)	C35—H35	0.9300
C8—C80	1.494 (3)	C36—H36	0.9300
C8A—N9	1.360 (2)	C80—F83	1.335 (3)
N9—C9A	1.315 (2)	C80—F82	1.343 (4)
C11—C12	1.369 (3)	C80—F81	1.343 (4)
C11—C16	1.387 (3)		
			
N9···F82	2.859 (3)	N9···C80	2.808 (3)
N9···F81	2.886 (2)		
			
C9A—N1—N2	111.17 (15)	C13—C12—H12	120.3
C9A—N1—C11	129.57 (17)	C14—C13—C12	120.0 (3)
N2—N1—C11	119.25 (15)	C14—C13—H13	120.0
C3—N2—N1	107.64 (16)	C12—C13—H13	120.0
N2—C3—C3A	110.47 (17)	C15—C14—C13	120.3 (2)
N2—C3—C31	120.29 (18)	C15—C14—H14	119.9
C3A—C3—C31	129.21 (18)	C13—C14—H14	119.9
C4—C3A—C9A	116.85 (17)	C14—C15—C16	121.0 (3)
C4—C3A—C3	138.44 (18)	C14—C15—H15	119.5
C9A—C3A—C3	104.65 (15)	C16—C15—H15	119.5
C3A—C4—C4A	118.50 (17)	C11—C16—C15	118.4 (3)
C3A—C4—H4	120.8	C11—C16—H16	120.8
C4A—C4—H4	120.8	C15—C16—H16	120.8
C4—C4A—C5	122.12 (17)	C36—C31—C32	118.8 (2)
C4—C4A—C8A	119.17 (16)	C36—C31—C3	121.0 (2)
C5—C4A—C8A	118.71 (18)	C32—C31—C3	120.2 (2)
C6—C5—C4A	121.01 (19)	C33—C32—C31	120.1 (3)
C6—C5—H5	119.5	C33—C32—H32	120.0
C4A—C5—H5	119.5	C31—C32—H32	120.0
C5—C6—C7	120.43 (18)	C34—C33—C32	120.9 (3)
C5—C6—H6	119.8	C34—C33—H33	119.6
C7—C6—H6	119.8	C32—C33—H33	119.6
C8—C7—C6	121.0 (2)	C35—C34—C33	119.2 (3)
C8—C7—H7	119.5	C35—C34—H34	120.4
C6—C7—H7	119.5	C33—C34—H34	120.4
C7—C8—C8A	120.35 (19)	C34—C35—C36	120.3 (3)
C7—C8—C80	120.32 (19)	C34—C35—H35	119.8
C8A—C8—C80	119.32 (17)	C36—C35—H35	119.8
N9—C8A—C8	118.39 (17)	C31—C36—C35	120.7 (3)
N9—C8A—C4A	123.15 (17)	C31—C36—H36	119.6
C8—C8A—C4A	118.47 (16)	C35—C36—H36	119.6
C9A—N9—C8A	114.56 (16)	F83—C80—F82	106.0 (2)
N9—C9A—N1	126.14 (16)	F83—C80—F81	106.3 (2)
N9—C9A—C3A	127.78 (16)	F82—C80—F81	106.12 (18)
N1—C9A—C3A	106.07 (16)	F83—C80—C8	112.35 (18)
C12—C11—C16	121.0 (2)	F82—C80—C8	112.9 (2)
C12—C11—N1	120.6 (2)	F81—C80—C8	112.6 (2)
C16—C11—N1	118.3 (2)	C80—F81—N9	73.15 (13)
C11—C12—C13	119.4 (2)	C80—F82—N9	74.18 (13)
C11—C12—H12	120.3		
			
C9A—N1—N2—C3	0.0 (3)	N2—N1—C9A—C3A	0.0 (3)
C11—N1—N2—C3	179.2 (3)	C11—N1—C9A—C3A	−179.2 (3)
N1—N2—C3—C3A	0.1 (3)	C4—C3A—C9A—N9	0.7 (4)
N1—N2—C3—C31	178.3 (2)	C3—C3A—C9A—N9	178.4 (3)
N2—C3—C3A—C4	176.8 (3)	C4—C3A—C9A—N1	−177.6 (2)
C31—C3—C3A—C4	−1.2 (6)	C3—C3A—C9A—N1	0.1 (3)
N2—C3—C3A—C9A	−0.1 (3)	C9A—N1—C11—C12	29.2 (4)
C31—C3—C3A—C9A	−178.1 (3)	N2—N1—C11—C12	−149.9 (3)
C9A—C3A—C4—C4A	−0.8 (4)	C9A—N1—C11—C16	−153.3 (3)
C3—C3A—C4—C4A	−177.5 (3)	N2—N1—C11—C16	27.6 (4)
C3A—C4—C4A—C5	−179.4 (3)	C16—C11—C12—C13	−0.9 (4)
C3A—C4—C4A—C8A	0.6 (4)	N1—C11—C12—C13	176.5 (3)
C4—C4A—C5—C6	−179.4 (3)	C11—C12—C13—C14	−0.3 (5)
C8A—C4A—C5—C6	0.6 (5)	C12—C13—C14—C15	1.4 (6)
C4A—C5—C6—C7	−0.9 (5)	C13—C14—C15—C16	−1.2 (6)
C5—C6—C7—C8	0.3 (5)	C12—C11—C16—C15	1.1 (4)
C6—C7—C8—C8A	0.5 (5)	N1—C11—C16—C15	−176.3 (3)
C6—C7—C8—C80	−178.5 (3)	C14—C15—C16—C11	0.0 (5)
C7—C8—C8A—N9	179.6 (3)	N2—C3—C31—C36	157.4 (3)
C80—C8—C8A—N9	−1.4 (4)	C3A—C3—C31—C36	−24.7 (4)
C7—C8—C8A—C4A	−0.7 (4)	N2—C3—C31—C32	−23.9 (4)
C80—C8—C8A—C4A	178.3 (3)	C3A—C3—C31—C32	153.9 (3)
C4—C4A—C8A—N9	−0.2 (4)	C36—C31—C32—C33	−0.3 (4)
C5—C4A—C8A—N9	179.8 (3)	C3—C31—C32—C33	−179.0 (3)
C4—C4A—C8A—C8	−179.9 (3)	C31—C32—C33—C34	0.6 (5)
C5—C4A—C8A—C8	0.1 (4)	C32—C33—C34—C35	−0.5 (5)
C8—C8A—N9—C9A	179.7 (2)	C33—C34—C35—C36	0.1 (6)
C4A—C8A—N9—C9A	0.1 (4)	C32—C31—C36—C35	−0.1 (5)
C8—C8A—N9—C80	0.7 (2)	C3—C31—C36—C35	178.5 (3)
C4A—C8A—N9—C80	−178.9 (3)	C34—C35—C36—C31	0.2 (6)
C8—C8A—N9—F82	−21.1 (2)	C7—C8—C80—F83	−1.4 (4)
C4A—C8A—N9—F82	159.3 (2)	C8A—C8—C80—F83	179.6 (3)
C8—C8A—N9—F81	23.3 (2)	C7—C8—C80—F82	−121.3 (3)
C4A—C8A—N9—F81	−156.3 (2)	C8A—C8—C80—F82	59.7 (3)
C8A—N9—C9A—N1	177.7 (2)	C7—C8—C80—F81	118.6 (3)
C8A—N9—C9A—C3A	−0.3 (4)	C8A—C8—C80—F81	−60.4 (3)
N2—N1—C9A—N9	−178.4 (3)	C7—C8—C80—N9	179.7 (3)
C11—N1—C9A—N9	2.4 (5)	C8A—C8—C80—N9	0.68 (19)

### Hydrogen-bond geometry (Å, º)

**Table:**

*D*—H···*A*	*D*—H	H···*A*	*D*···*A*	*D*—H···*A*
C12—H12···N9	0.93	2.50	3.042 (3)	118
C7—H7···F83	0.93	2.35	2.692 (3)	102
C36—H36···F82^i^	0.93	2.56	3.357 (3)	144
C13—H13···*Cg*4^ii^	0.93	2.92	3.729 (4)	146
C33—H33···*Cg*4^iii^	0.93	3.03	3.697 (4)	130

Symmetry codes: (i) −*x*, *y*+1/2, −*z*+1; (ii) −*x*, *y*+1/2, −*z*+2; (iii) −*x*−1, *y*−1/2, −*z*+1.
